# Study on phytotoxicity evaluation and physiological properties of nicosulfuron on sugar beet (*Beta vulgaris* L.)

**DOI:** 10.3389/fpls.2022.998867

**Published:** 2022-10-11

**Authors:** Longfeng Wang, Muhammad Riaz, Baiquan Song, Xin Song, Wengong Huang, Xiaoshan Bai, Xiaoyu Zhao

**Affiliations:** ^1^ College of Advanced Agriculture and Ecological Environment, Heilongjiang University, Harbin, China; ^2^ Research Institute of Economic Crops, Xinjiang Academy of Agricultural Sciences, Urumqi, Xinjiang, China; ^3^ State Key Laboratory of Conservation and Utilization of Subtropical Agro-biore Sources, Root Biology Center, College of Natural Resources and Environment, South China Agricultural University, Guangzhou, China; ^4^ Safety and Quality Institution of Agricultural Products, Heilongjiang Academy of Agricultural Sciences, Harbin, China

**Keywords:** herbicide, phytotoxicity, lethal dose, chlorophyll fluorescence, oxidative defense

## Abstract

Nicosulfuron is an herbicide widely used in corn fields. In northeast China, sugar beet is often planted adjacent to corn, resulting in frequent phytotoxicity of nicosulfuron drift in sugar beet fields. This study was conducted by spraying nicosulfuron to assess the phytotoxicity and clarify the mechanism of nicosulfuron toxicity on sugar beet. The results showed that nicosulfuron impaired growth and development by reducing photosynthetic capacity and disrupting antioxidant systems at a lethal dose of 81.83 g a.i. ha^–1^. Nicosulfuron damaged the function of photosynthetic system II (PSII), lowered photosynthetic pigment content, and inhibited photosynthetic efficiency. Compared with the control, the electron transfer of PSII was blocked. The ability of PSII reaction centers to capture and utilize light energy was reduced, resulting in a weakened photosynthetic capacity. The maximum net photosynthetic rate (Amax), light saturation point (LSP), and apparent quantum yield (AQY) decreased gradually as the nicosulfuron dose increased, whereas the light compensation point (LCP) and dark respiration (Rd) increased. Nicosulfuron led to reactive oxygen species (ROS) accumulation in sugar beet leaf, a significant rise in malondialdehyde (MDA) content, electrolytic leakage (EL), and considerable oxidative damage to the antioxidant system. This study is beneficial for elucidating the effects of nicosulfuron toxicity on sugar beet, in terms of phytotoxicity, photosynthetic physiology, and antioxidative defense system.

## 1 Introduction

Weeds are an important limiting factor in agricultural production (Ghosh et al., 2020; Skalicky et al., 2020). Herbicide application is a common means of weed control in agricultural production, but improper use can easily cause phytotoxicity. In recent years, herbicide has attracted widespread attention. Studies have shown that crops such as rice (Bellaloui et al., 2006), soybean (Brown et al., 2009), peanut (Koger et al., 2010), sorghum (Steppig et al., 2017), maize (Egan et al., 2017), wheat (Wiersma and Durgan, 2018) and melon (Xu et al., 2018) have been infested with herbicide toxicity, affecting growth and crop yield.

Nicosulfuron is widely used in corn fields because of its fast-acting, strong persistence and high safety. It is an Acetolactate Synthase (ALS) inhibitor herbicide that inhibits ALS enzyme activity in sensitive plants, thereby inhibiting the formation of branched-chain multiple amino acids (Wright et al., 2017). As a result, plants affected by nicosulfuron will eventually stop growing, or even die. Field weed resistance and the relentless pursuit of crop yield have resulted in an increase in the use of nicosulfuron in agricultural production year after year. Herbicide phytotoxicity not only occurs in crops but also causes phytotoxicity to neighboring crops due to herbicide drift of droplets formed during herbicide spraying (Meloni and Bolzón, 2021).

Sugar beet (*Beta vulgaris* L.), a widespread sugar crop in temperate climates, meets about 20% of the global sugar demand (Song et al., 2022). At the same time, sugar beet is a susceptible crop to herbicides. It is often damaged by herbicide drift from adjacent field crops (Li et al., 2021). Corn and sugar beet are frequently cultivated adjacent in northeast China. Nicosulfuron toxicity is a common phenomenon in local production areas and is an important cause of sugar beet yield decline (Wang et al., 2009; Ellis and Miller, 2010). Since corn is a monocotyledonous plant, nicosulfuron is commonly used in corn fields to control dicotyledonous weeds. Because of this, nicosulfuron drift is more harmful to sugar beet that grows next to corn fields (Li et al., 2017).

Under herbicide stress, plants usually produce large amounts of reactive oxygen species (ROS), leading to oxidative stress in plants. The surge of ROS activates the plant’s antioxidant system, which allows the plant to scavenge excess ROS (Jervekani et al., 2018; Li et al., 2022b). At the same time, plants are also able to respond to herbicide stress by regulating hormonal activity and promoting or inhibiting the formation of key metabolites. Therefore, it is common to mitigate herbicide toxicity in crops by application of plant hormones (Li et al., 2022a).

The effects of Herbicide toxicity stress on crop growth parameters, photosynthetic properties, and antioxidant systems have received extensive attention, including wheat (Yadav et al., 2019; Feng et al., 2021), maize (Wang et al., 2018; Wang et al., 2021a; Sun et al., 2022) and black bean (Meloni and Bolzón, 2021). However, fewer studies have been reported on sugar beet toxic symptoms and photosynthetic physiology under herbicide toxicity. In particular, the response of sugar beet under nicosulfuron stress in terms of physiology, photosynthetic system and the antioxidant system is not clear. Consequently, a pot experiment was conducted to explore the phytotoxic effects of nicosulfuron on sugar beet, to provide a reference for assessing herbicide phytotoxicity and addressing nicosulfuron drift damage on sugar beet.

## 2 Materials and methods

### 2.1 Experimental material

Sugar beet variety KWS1176 was provided by Seed Co., Ltd. (Germany). Qingdao Hansen Bioscience Co., Ltd. supplied the 24% nicosulfuron oil suspension. The soil type is black soil with the following initial properties, pH: 6.64; bulk density: 1.26 g cm^–3^; organic matter content: 21.23 g kg^–1^; alkali-hydrolyzable N: 122.64 mg kg^–1^; available P: 46.30 mg kg^–1^; available K: 330.92 mg kg^–1^.

### 2.2 Experimental design

The experiment was carried out in a greenhouse at Heilongjiang University, China. The test soil was filled with 0.073 g kg^–1^ of urea, 0.078 g kg^–1^ of phosphate diamine, and 0.095 g kg^–1^ of potassium sulfate in polyethylene plastic pots (300 g per pot) and poured with 45 mL of distilled water. Each pot was sown with 3 sugar beet seeds, and covered with 100 g of soil. The seedlings were cultivated in a greenhouse under natural light with a light intensity of 138 mol m^-2^ s^-1^, 14 h of light per day, 25°C/20°C (day/night), and 50–60% relative humidity. One plant was left in each pot after one week of cultivation.

The recommended dose of nicosulfuron in the corn field was 60 g a.i. ha^–1^. Considering the herbicide over-application in agricultural production, the nicosulfuron doses of the five treatment groups were designated 1/100, 1/10, 1/3, 1, and 2 times the recommended dose in the field, noted as N0.6, N6, N20, N60, N120. Water was sprayed as a control group (CK) and each treatment was replicated six-time. The sugar beet seedlings were sprayed with various concentrations of nicosulfuron solution once the second pair of sugar beet leaves were utterly extended. Control treatments were sprayed with distilled water.

### 2.3 Measurement of phytotoxicity index and physiological properties

Phytotoxicity index, growth indexes, photosynthetic parameters, and fluorescence parameters were measured within 20 days after being treated with nicosulfuron. On 20 DAT (days after treatment), samples of the second pair of true leaves of sugar beet were taken and stored at −20°C to determine physiological indicators.

#### 2.3.1 Determination of growth parameters

SPAD values of the second pair of true leaves of sugar beet were measured using SPAD chlorophyll meter (Minolta SPAD-502Plus, Tokyo, Japan). The plant height, leaf length and leaf width of the second pair of true leaves in the natural state of the sugar beet were measured using a straightedge. To collect the samples, the beets were removed from the pots, cleaned of root soil, and placed flat on a glass plate. The plants extended naturally and the length of the underground part of the plants was recorded as root length with a straightedge. The root thickness was measured with vernier calipers. Leaf area was calculated from the leaf area index (Hoffmann and Blomberg, 2004). The above- and below-ground parts were split with scissors and the fresh weight of the plants was determined separately. Beets were killed in an oven at 120°C for 2 h, dried at 80°C to a constant weight, and weighed for dry weight after natural cooling.

#### 2.3.2 Calculation of phytotoxicity index and dose-fresh weight response curve

The phytotoxicity index was calculated based on the phytotoxicity grade (Dai et al., 2017) (**Table 1**).

**Table 1:** Classification standard of phytotoxicity grades.

Phytotoxicity grade	Description of phytotoxicity symptoms
0	Control treatment
1	Seedlings’ height and leaf color slightly different from the control
2	Seedlings were slightly deformed, lower in height than the control
3	Seedlings were shorter, with thicker stalks, slightly thicker leaves, and yellow color
4	Seedlings stopped growth. Seedlings were deformed and stiff or the whole leaf was yellow and dead.
5	Seedlings death

To get the dose-fresh weight response curve, a three-parameter log-logistic model in R Studio was utilized to perform regression analysis on the dose-fresh weight response data (Stevan et al., 2007). The effective herbicide dosage that resulted in a 50% growth reduction (GR_50_) was determined.

#### 2.3.3 Determination of leaf photosynthetic parameters

The photosynthetic pigment content was determined using the ethanol method (Arnon, 1949). The net photosynthetic rate (P_n_), stomatal conductance (G_s_), transpiration rate (T_r_), and intercellular CO_2_ concentration (C_i_) of the second pair of true leaves of sugar bee were determined with a portable photosynthesis instrument, TARGAS-1 (Deligiosa et al., 2019). The investigations were run during 9:00-11:00 AM under the photosynthetically active radiation (PAR) level of 250 µmol m^–2^ s^–1^. The PAR levels of 1500, 1200, 800, 600, 400, 300, 200, 100, and 0 μmol m^–2^ s^–1^ were measured to get the P_n_-light curve, G_s_-light curve, T_r_-light curve and C_i_-light curve. The non-rectangular hyperbola model was utilized to calculate photosynthetic parameters, including the maximum net photosynthetic rate (Amax), light compensation point (LCP), light saturation point (LSP), apparent quantum yield (AQY), and dark respiration (Rd) (Ye et al., 2014).

#### 2.3.4 Determination of chl a fluorescence parameters

The chl a fluorescence transient (OJIP transient) of the second fully expanded sugar beet leaf under different treatments was determined using Pocket PEA continuous excitation fluorimeter (Handy, UK). The initial fluorescence (F_O_) was set as O (50 μs), K (300 μs), J (2 ms) and I (30 ms) are the intermediates (F_K_, F_J_ and F_I_, respectively) and P (1000 ms) as the maximum fluorescence (F_m_). The original (without normalization) chl a fluorescence intensity (F_t_) curves were plotted. The original OJIP transients were double normalized between the two fluorescence extreme O (F_O_) and P (F_m_) phases and the variable fluorescence between OP expressed as V_O–P_ was determined. The difference in transients (ΔV_O–P_) concerning a reference was calculated. Further, the chl a fluorescence transients were double normalized between F_O_ and F_J_ expressed as V_O–J_ and the difference between transients expressed as ΔV_O–J_ was determined.

Maximal Photochemical Efficiency of PSII (F_V_/F_m_), performance index on absorption basis (PI_abs_), electron transport flux per reaction center (RC) (ET_O_/RC), dissipated energy flux per RC (DI_O_/RC), absorption flux per RC (ABS/RC), dissipated energy flux per CS (DI_O_/CS_M_), electron transport flux per CS (ET_O_/CS_M_), section absorption flux per CS (ABS/CS_M_) were measured based on the above fluorescence parameters as reported by Strasser et al. (1995).

#### 2.3.5 Determination of physiological indicators

Physiological indicators were determined using the second pair of true leaves of sugar beet from the stored samples. The content of superoxide anion $\left({\text{O}}_{2}^{-}\right)$ was measured as reported by Zhang et al. (2007). The hydrogen peroxide (H_2_O_2_) content was measured as reported by Wang et al. (2021b). The malondialdehyde (MDA) content was measured by the thiobarbituric acid reaction (Dhindsa and Matowe, 1981). Electrolytic leakage (EL) was measured by a multi-parameter water quality analyzer (DZS-706-A) according to Belkhadi et al. (2010).

The activities of superoxide dismutase (SOD), peroxidase (POD), catalase (CAT), and ascorbate peroxidase (APX) were determined according to the approach of NBT reduction (Giannopolites and Ries, 1977), guaiacol method (Chance and Maehly, 1955), UV absorption method (Khorram et al., 2016), and the way of Jiang and Zhang (2001), respectively.

### 2.4 Data analysis

The data were analyzed by one-way ANOVA and Duncan’s method, and differences across groups were assessed. All data were expressed as ‘Means ± SD’. IBM SPSS Statistics 26 (SPSS Inc., Chicago, IL, USA) were applied for data analysis. Origin 2018 (OriginLab, Northampton, 210 MA, USA) was employed to draw graphs.

## 3 Results

### 3.1 Effects of nicosulfuron on the growth parameters of sugar beet

**Figure 1:**
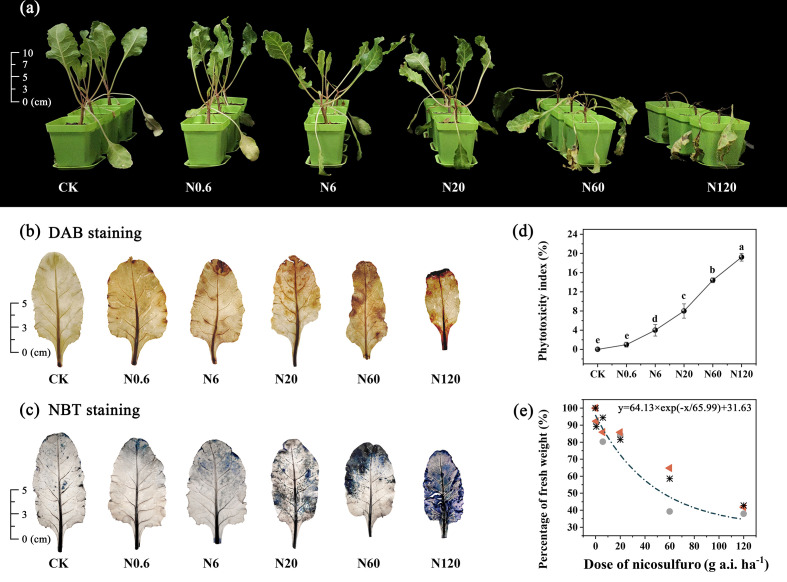
Effects of nicosulfuron on visible symptoms of phytotoxicity on sugar beet. The growth of sugar beet **(A)**, DAB staining **(B)**, NBT staining **(C)**, phytotoxicity index **(D)**, and dose-fresh weight response curve **(E)** in sugar beet on 20 DAT with different doses of nicosulfuron. Triangles, circles and asterisks represent different repetitions. Data with the different letters indicate significant differences between different doses of nicosulfuron drift (n = 6, p < 0.05).

The symptoms of phytotoxicity appeared on 4 DAT. On 20 DAT, sugar beet stopped growth when the dose of nicosulfuron reached 20 g a.i. ha^–1^. The plants were deformed, and yellow spots on leaves were obvious. Sugar beet seedlings were wilted and deformed at a recommended dosage of 60 g a.i.ha^–1^. The plant mortality rate was 60%, with the growing point as the starting point and extending upward to the petiole blackened. All plants died at 120 g a.i. ha^–1^ (**Figure 1A**). As the dose of nicosulfuron increased, the area of sugar beet leaves was enlarged and damage was visible (**Figures 1B, C** **)**. The phytotoxicity index showed a remarkable difference between treatment groups and CK at 6 g a.i. ha^–1^ and above (*p* < 0.05) (**Figure 1D**).

The dose-fresh weight response regression equation for nicosulfuron was calculated as y = 64.13 × exp (–x/65.99) + 31.63 (y represents the percentage of fresh weight in each treatment to the fresh weight in the control group and x represents the dose of nicosulfuron). The lethal dose GR_50_ value was 81.83 g a.i. ha^–1^, which was higher than the recommended field dose (60 g a.i. ha^–1^) by 36.38% (**Figure 1E**).

The biomass of shoot and root were reduced with expanding doses of nicosulfuron. The shoot biomass were more affected than the root. There was a remarkable difference in shoot DW compared with CK when the dose reached 6 g a.i. ha^–1^, with a 45% reduction (*p* < 0.05). At this dose, the dry weight of the shoot did not change significantly, which was only 7.69% lower than the control (**Table 2**).

**Table 2:** Effects of nicosulfuron on biomass of sugar beet.

Treatment	Shoot		Root		Root-shoot ratio
	FW (g plant^–1^)	DW (g plant^–1^)	FW (g plant^–1^)	DW (g plant^–1^)
CK	4.31 ± 0.42a	0.78 ± 0.02a	0.25 ± 0.02a	0.15 ± 0.03a	0.09 ± 0.01a
N0.6	3.91 ± 0.33a	0.77 ± 0.04a	0.23 ± 0.02a	0.13 ± 0.01a	0.07 ± 0.01ab
N6	3.76 ± 0.64a	0.72 ± 0.04ab	0.22 ± 0.06ab	0.10 ± 0.03a	0.05 ± 0.02bc
N20	3.63 ± 0.28a	0.69 ± 0.03b	0.19 ± 0.01ab	0.09 ± 0.06a	0.05 ± 0.01bc
N60	2.33 ± 0.59b	0.57 ± 0.01c	0.16 ± 0.03b	0.11 ± 0.03a	0.04 ± 0.01c
N120	1.87 ± 0.25b	0.60 ± 0.04c	0.07 ± 0.01c	0.11 ± 0.02a	0.03 ± 0.01c

FW, fresh weight; DW, dry weight. Data with the different letters indicate significant differences between different doses of nicosulfuron drift (n = 6, p < 0.05).

**Figure 2:**
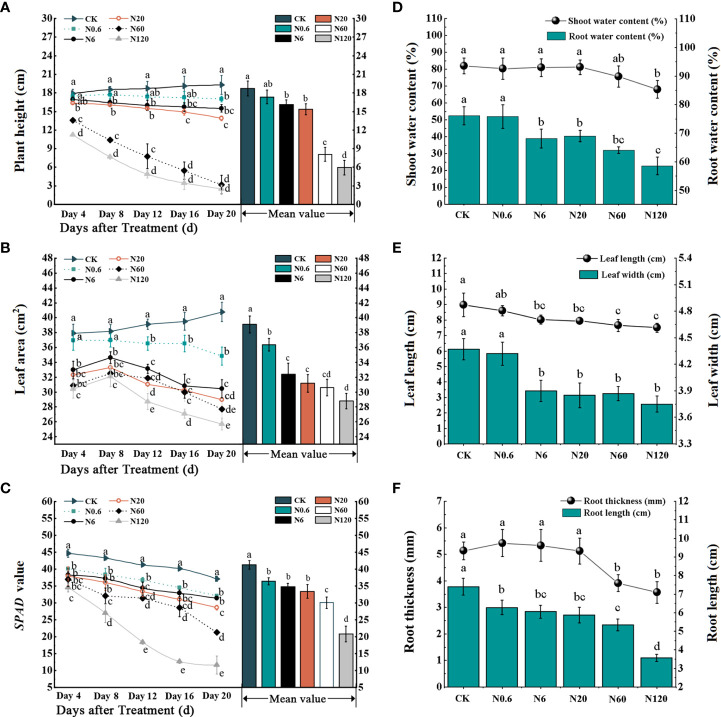
Effects of nicosulfuron on the growth parameters of sugar beet. Plant height **(A)**, leaf area **(B)**, *SPAD* value **(C)**, shoot water content, root water content **(D)**, leaf width, leaf length **(E)**, root width and root length **(F)** in sugar beet with different doses of nicosulfuron. Data with the different letters indicate significant differences between different doses of nicosulfuron drift (n = 6, *p* < 0.05).

All plant growth parameters were significantly reduced with increasing dose, such as plant height, leaf area, and *SPAD* value. The plant height, leaf area, and *SPAD* value were significantly different from the control at 20 DAT as the dose reached 0.6 g a.i. ha^–1^ (*p* < 0.05) (**Figures 2A, B, C** **)**. There was a remarkable inhibition in shoot water content, leaf length, leaf width, and root length at 6 g a.i. ha^–1^ compared to the control, 10.45%, 10.63%, 10.76% and 18.24% (*p* < 0.05) (**Figures 2D, E, F** **)**.

### 3.2 Effects of nicosulfuron on the photosynthetic parameters of sugar beet leaf

**Figure 3:**
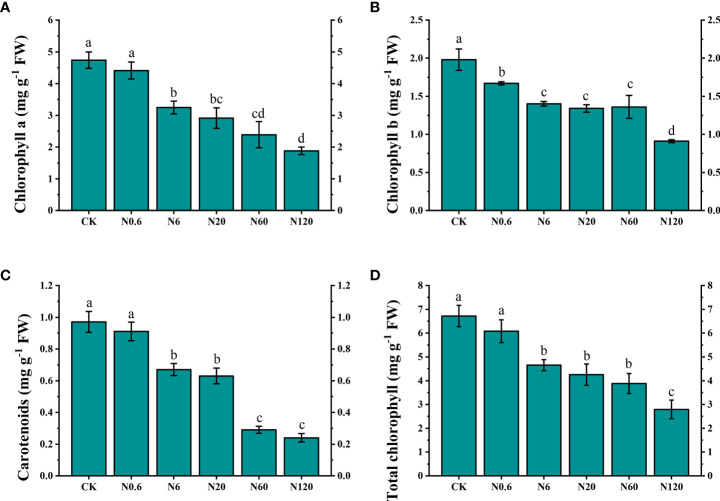
Effects of nicosulfuron on the photosynthetic pigment of sugar beet leaf. Chlorophyll a **(A)**, chlorophyll b **(B)**, carotenoids **(C)** and total chlorophyll **(D)** in sugar beet on 20 DAT with different doses of nicosulfuron. FW: fresh weight. Data with the different letters indicate significant differences between different doses of nicosulfuron drift (n = 6, *p* < 0.05).

The content of photosynthetic pigment was reduced with increasing doses of nicosulfuron. When the dose of nicosulfuron reached 6 g ai ha^–1^, the content of chlorophyll a, b, and carotenoids were decreased by 31.43%, 29.29% and 31.36%, compared to CK, respectively (*p* < 0.05). When the dose reached the highest dose in this study (120 g a.i. ha^–1^), the content of carotenoids and total chlorophyll decreased by 75.75% and 58.48% (**Figure 3**).

**Figure 4:**
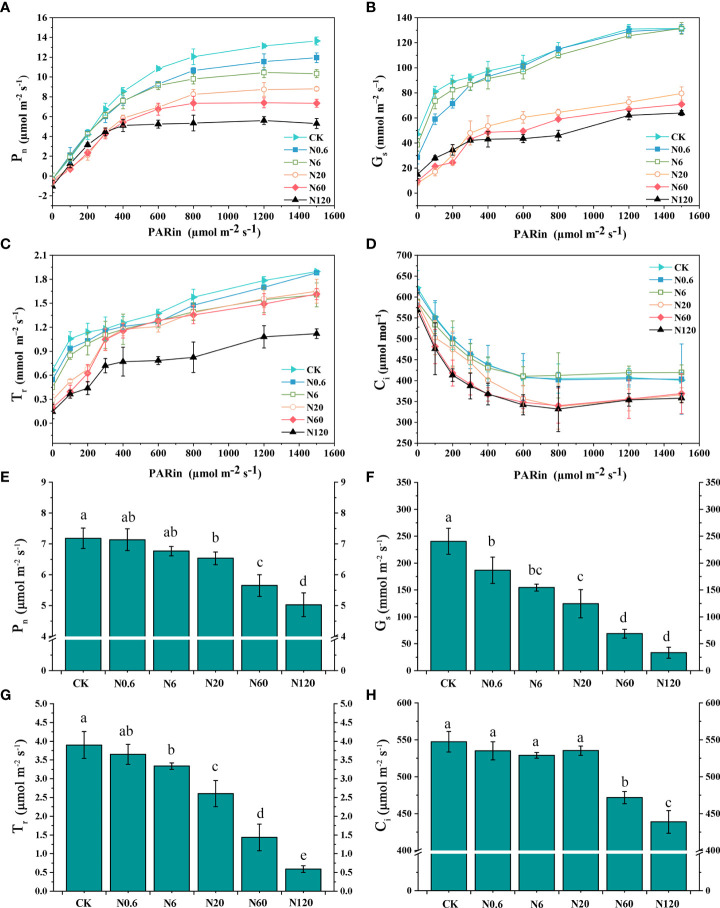
Effects of nicosulfuron on the gas exchange parameters of sugar beet leaf. P_n_-light curve **(A)**, G_s_-light curve **(B)**, T_r_-light curve **(C)**, C_i_-light curve **(D)**, Net photosynthetic rate (P_n_) **(E)**, stomatal conductance (G_s_) **(F)**, transpiration rate (T_r_) **(G)** and intercellular CO_2_ concentration (C_i_) **(H)** in sugar beet on 20 DAT with different doses of nicosulfuron. Data with the different letters indicate significant differences between different doses of nicosulfuron drift (n = 6, *p* < 0.05).

The P_n_ of sugar beet leaf showed a linear enhancement trend with light intensity as PAR increased when the PAR was under 400 μmol m^–2^ s^–1^. After that, the increase in P_n_ slowed under each treatment as PAR continued to grow. Under different doses of nicosulfuron treatment, the changing pattern of the P_n_-light curve began to differ as the PAR was over 400 μmol m^–2^ s^–1^. The highest P_n_-light curve changes were observed in CK treatment and the lowest in N120 treatment. As the PAR reached 1200 μmol m^–2^ s^–1^, P_n_ gradually saturated (**Figure 4A**). Both G_s_ and T_r_ showed an upward trend with increased PAR and nicosulfuron dose while C_i_ declined (**Figures 4B, C, D** **)**.

The inhibitory effects of nicosulfuron on P_n_, G_s_, T_r_ and C_i_ increased with increased doses of nicosulfuron. There were remarkable differences in P_n_, G_s_, T_r_, and C_i_ at 60 g a.i. ha^–1^ as compared with CK (*p* < 0.05) (**Figures 4E, G, H** **)**. Only the difference in G_s_ reached significance at the lowest dose of nicosulfuron (0.6 g a.i. ha^–1^) in the study, with a reduction of 22.39% as compared with CK (*p* < 0.05) (**Figure 4F**).

The Amax, LSP and AQY gradually decreased with increasing nicosulfuron dose as compared with CK. Specifically, the differences in Amax, LSP and AQY reached significance when the dose reached 0.6 g a.i. ha^–1^ were 12.45%, 8.05%, and 15.79% lower than the control (*p* < 0.05). The LCP and Rd increased with increasing nicosulfuron dose. The difference between Rd and the control was significant when the dose was over 0.6 g a.i. ha^–1^, and the Rd increased by 35.14% at 0.6 g a.i. ha^–1^ (*p* < 0.05) (**Table 3**).

**Table 3:** Effects of nicosulfuron on the P_n_-PAR curve parameters of sugar beet leaf.

Treatments	Amax (µmol m^–2^ s^–1^)	LCP (µmol m^–2^ s^–1^)	LSP (µmol m^–2^ s^–1^)	AQY	Rd (µmol m^–2^ s^–1^)
CK	13.57 ± 0.01a	12.60 ± 1.42a	1433.71 ± 76.26a	0.038 ± 0.001a	0.37 ± 0.03a
N0.6	11.88 ± 0.07b	15.94 ± 1.61a	1318.94 ± 12.43b	0.032 ± 0.002b	0.50 ± 0.05b
N6	10.58 ± 0.01c	20.87 ± 1.56a	1166.65 ± 9.81c	0.032 ± 0.001b	0.62 ± 0.09bc
N20	9.03 ± 0.02d	32.40 ± 3.66b	1205.10 ± 9.78c	0.030 ± 0.001c	0.65 ± 0.06bc
N60	7.79 ± 0.01e	34.56 ± 1.76bc	1082.99 ± 2.64d	0.022 ± 0.002d	0.72 ± 0.14cd
N120	5.69 ± 0.03f	42.88 ± 5.42c	915.21 ± 3.74e	0.020 ± 0.003d	1.17 ± 0.04d

Amax, maximum net photosynthetic rate; LSP, light saturation point; AQY, apparent quantum yield; Rd, dark respiration. Data with the different letters indicate significant differences between different doses of nicosulfuron drift (n = 6, p < 0.05).

### 3.3 Effects of nicosulfuron on the chl a fluorescence parameters of sugar beet leaf

**Figure 5:**
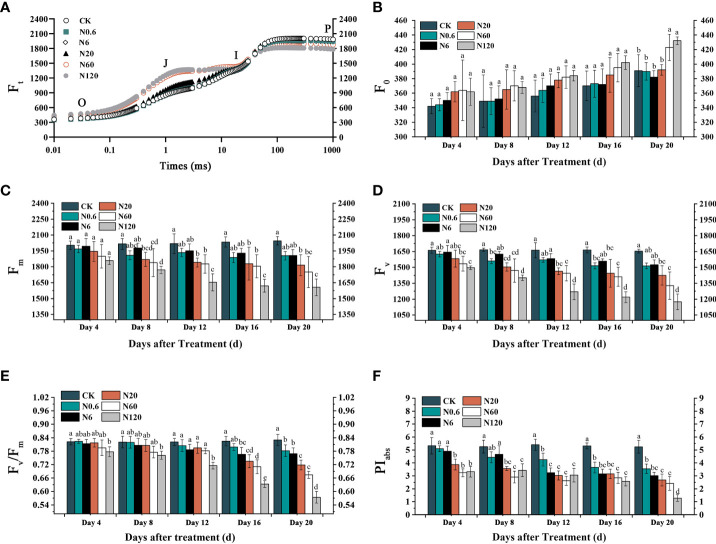
Effects of nicosulfuron on the PSII activity of sugar beet leaf. OJIP transient on 20 DAT **(A)**, F_O_ **(B)**, F_m_ **(C)**, F_v_ **(D)**, F_v_/F_m_ **(E)** and PI_abs_ **(F)** in sugar beet with different doses of nicosulfuron. Data with the different letters indicate significant differences between different doses of nicosulfuron drift (n = 6, *p* < 0.05).

On the OJIP transient, fluorescence intensity at the O point showed an upward trend as the doses of nicosulfuron treatment increased. In contrast, it showed the opposite at the P point. (**Figure 5A**). The effects of nicosulfuron on F_m_ and F_v_ were more pronounced compared to F_O_. At 6 g a.i. ha^–1^, F_m_ showed remarkable differences at 8 DAT (**Figures 5B, C, D** **)**. The variations of F_v_/F_m_ and PI_abs_ were decreased with increasing doses. On 20 DAT, F_v_/F_m_ and PI_abs_ significantly decreased by 18.75% and 53.86% at 60 g a.i. ha^–1^ (*p* < 0.05) (**Figures 5E, F** **)**.

**Figure 6:**
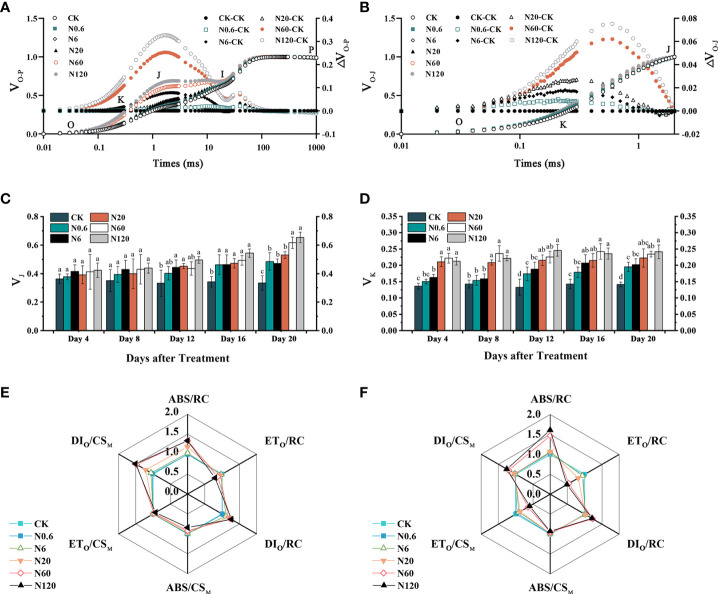
Effects of nicosulfuron on the photosynthetic energy of sugar beet leaf. V_O-P_, ΔV_O-P_ curves on 20 DAT **(A)**, V_O-J_, ΔV_O-J_ curves on 20 DAT **(B)**, V_J_ **(C)**, V_K_ **(D)**, energy distribution parameters on 4 DAT **(E)** and 20 DAT **(F)** in sugar beet leaf after treatment with different doses of nicosulfuron. Data with the different letters indicate significant differences between different doses of nicosulfuron drift (n = 6, *p* < 0.05).

The V_J_ and V_K_ of sugar beet leaf significantly lowered under nicosulfuron toxicity. The V_K_ was more remarkably affected than V_J_ (**Figures 6A, B** **)**. At 20 DAT, V_J_ and V_K_ increased significantly by 84.20% and 96.33% at 60 g a.i. ha^–1^ contrasted with CK (*p* < 0.05) (**Figures 6C, D** **)**. The ABS/CS_M_ and ET_O_/CS_M_ of sugar beet leaf declined with the increase of nicosulfuron dose while DI_O_/CS_M_ increased significantly. The DI_O_/RC and ABS/RC increased, while ET_O_/RC reduced (**Figure 6E**). The trends of each light energy absorption and distribution parameter on 20 DAT were consistent with those of the 4 DAT, but the changes were significantly greater than those of the 4 DAT (**Figure 6F**).

### 3.4 Effects of nicosulfuron on the physiological indicators of sugar beet leaf

**Figure 7:**
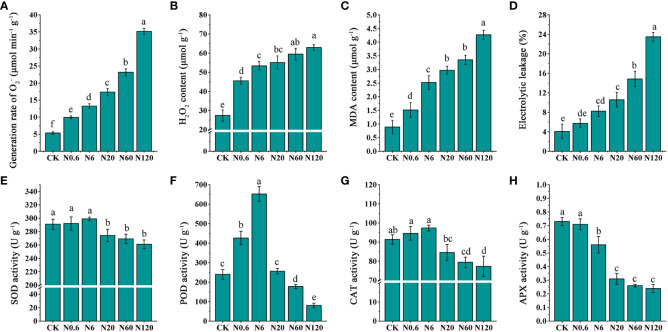
Effects of nicosulfuron the on physiological indicators of sugar beet leaf. The generation rate of ${\text{O}}_{2}^{-}$ **(A)**, H_2_O_2_ content **(B)**, malondialdehyde (MDA) content **(C)**, electrolytic leakage (EL) **(D)**, SOD activity **(E)**, POD activity **(F)**, CAT activity **(G)**, and APX activity **(H)** in sugar beet on 20 DAT with different doses of nicosulfuron. Data with the different letters indicate significant differences between different doses of nicosulfuron drift (n = 6, *p* < 0.05).

An increased dose of nicosulfuron enhanced the generation rate of ${\text{O}}_{2}^{-}$ , the contents of H_2_O_2_, MDA and EL in sugar beet leaf. When the dose reached 0.6 g a.i. ha^–1^, the generation rate of ${\text{O}}_{2}^{-}$ and H_2_O_2_ contents increased significantly by 84.73% and 65.96% (*p* < 0.05) (**Figures 7A, B** **)**. The differences in MDA content and EL reached significant amounts at 6 g a.i. ha^–1^, increasing by 183.15% and 102.46% (*p* < 0.05) (**Figures 7C, D** **)**.

The SOD, POD and CAT activities were enhanced first and afterward lowered, reaching a peak at 6 g a.i. ha^–1^ as the dose of nicosulfuron increased, while the APX activity decreased gradually. The difference in SOD and CAT activities was significantly contrasted with CK lowered by 5.74% and 7.44% at 20 g a.i. ha^–1^ (*p* < 0.05) (**Figures 7E, G** **)**. The POD activity was in an upward trend at 0.6 g a.i. ha^–1^, which significantly increased 77.78% compared to the control (*p* < 0.05). And it was on the decline at 60 g a.i. ha^–1^, with a remarkable reduction of 25.93% contrasted with the control (*p* < 0.05) (**Figures 7F**). APX activity was significantly lowered by 23.29% contrasted with CK at 6 g a.i. ha^–1^ (*p* < 0.05) (**Figures 7H**).

## 4 Discussion

### 4.1 Nicosulfuron phytotoxicity repressed the growth of sugar beet seedlings

Growth parameters are the most visible indicator of the degree of crop phytotoxicity in a stress condition. The most commonly used method for describing herbicide phytotoxicity is a simple and subjective visual estimation of the observed crop injury (Weber et al., 2017). Nicosulfuron can be harmful to plants, leading to the inhibition of plant growth indicators (Wang et al., 2021b). Under the influence of nicosulfuron at the recommended dose of nicosulfuron in the field (60 g a.i. ha^–1^), plants showed the symptoms of phytotoxicity on 4 DAT. On 20 DAT, plants were deformed with blackened growing points and yellow leaves. Biomass was significantly suppressed, and the mortality rate was 60%. It was noteworthy that the symptoms were more pronounced on new leaves than on old leaves. This might be because nicosulfuron was a systemic herbicide that stems, leaves, and roots can taken up. The new leaves were young and metabolically active, so they were more susceptible to damage by such herbicides (Rey Caballero et al., 2016).

The phytotoxicity degree of nicosulfuron to plants depends on the dose. The range of variation in GR_50_ for different plants ranges from 0.95 to 169.93 g a.i. ha^–1^ (Xu et al., 2018), which indicates that the resistance to nicosulfuron varies widely among different plants. The reason is that the resistance of nicosulfuron depends mainly on the plant’s genetic material. There are differences in resistance even among different varieties of the same plant (Choe and Williams, 2020). In this experiment, the GR_50_ was 81.83 g a.i. ha^–1^ of sugar beet, which was close to the response of cocklebur (*X. strumarium* L.) to nicosulfuron (Božić et al., 2015). The GR_50_ of nicosulfuron on sugar beet increased by 36.38% of the recommended field dose of nicosulfuron (60 g a.i. ha^–1^). In comparison, the total drift of nicosulfuron was generally less than 25% of the total applied dose in agricultural production (Wang et al., 2009). So, the toxic effects of nicosulfuron drift on sugar beet were usually not deadly, even considering the over-application of nicosulfuron in agriculture.

### 4.2 Nicosulfuron inhibited photosynthetic performance in sugar beet

In this study, the contents of chlorophyll and carotenoid were significantly reduced by nicosulfuron toxicity. This might be due to the over-production of reactive oxygen species (ROS) inhibiting the photochemical activity of chloroplasts and blocking the formation of photosynthetic pigment. Another reason could be the degradation of chlorophyll due to cell damage caused by the accumulation of ROS. ALS inhibitors induced a reduction in P_n_ after being treated in plants according to numerous studies (Orcaray et al., 2010), which were consistent with the findings of this study. The reduction of P_n_ in sugar beet leaf by nicosulfuron toxicity was similar to the change in photosynthetic pigment content, which indicated that the decrease in photosynthetic pigment is one of the essential reasons for the decrease in P_n_. The stomata of plant leaf control gas exchange which can further affect photosynthetic capacity by limiting water loss and controlling CO_2_ uptake, affecting T_r_ and C_i_ (Hetherington and Woodward, 2003; Geiger et al., 2009). In this study, G_s_ gradually decreased with increasing doses, which led to a decrease in T_r_. Notably, C_i_ also showed a decreasing trend, suggesting that photosynthesis capacity might be limited by stomatal and non-stomatal factors (Singh et al., 2013).

Photosynthetic light-response curves can determine the extent to which the photosynthetic efficiency of plants is affected by environmental change. It is shown that nicosulfuron significantly suppressed the Amax, LSP and AQY, while the LCP, was increased. This indicated that the photosynthetic efficiency of sugar beet was significantly reduced in response to light environment change. This might be due to the fact that phytotoxicity reduced the pigment-protein complexes that absorb and convert light energy in sugar beet, resulting in a reduced ability of sugar beet to utilize both strong and weak light (He et al., 2018). The Rd of sugar beet leaf was significantly higher under nicosulfuron poisoning conditions, probably due to the inhibition of assimilate transport in sugar beet. Increased Rd consumed the excess assimilation accumulated in the leaves and slowed the inhibition of photosynthesis (Pavithra et al., 2020). This indicated that sugar beet adapted to the toxicity mainly by promoting respiration.

### 4.3 Nicosulfuron inhibited PSII activity and photosynthetic energy

By blocking the electron transport chain in chloroplasts, ALS inhibitors can damage the structure and function of the photosynthetic system II. F_v_/F_m_ and PI_abs_ are considered the most common indicators to characterize PSII reaction center activity (Yi et al., 2016). The most sensitive fluorescence parameter to different stress treatments was the PI_abs_. It is used to quantify the overall photosynthetic performance of the sample. The reduction of F_v_/F_m_ and PI_abs_ in this study demonstrated that nicosulfuron remarkably repressed the PSII reaction center activity of sugar beet leaf. The restraint extent was connected with nicosulfuron dose, similar to the investigation by Zhang et al. (2018). Compared with CK, both V_K_ and V_J_ increased to different degrees with increasing doses of nicosulfuron. The enhancement in V_J_ demonstrated the electron transfer process from Q_A_ to Q_B_ is blocked, which leads to a large accumulation of ${\text{Q}}_{A}^{-}$ , a typical inhibition of the PSII receptor side. The increase in V_K_ indicated that the PSII electron donor side of the oxygen-evolving complex OEC was destroyed (Strasser, 1997). This was consistent with the response of mulberry (Liu et al., 2018) and alfalfa (Guo et al., 2020) under herbicide stress. The most significant change in each characteristic point of the OJIP transient was the elevation of the J point. From this, we conclude that the PSII receptor side of sugar beet plants is more sensitive to nicosulfuron toxicity.

Under phytotoxic stress, plants often improve adaptation by adjusting energy distribution (Arthaud et al., 2021). With the increase of nicosulfuron dose, the variation of ABS/CS_M_ was decreased. This indicated that nicosulfuron caused inactivation of partial reaction centers in sugar beet leaf on the one hand and also damaged antenna pigment-protein which then resulted in a decrease in the amount of captured light energy and the reduction of ET_O_/CS_M_. The experiment showed a reduction in DI_O_/CS_M_ with nicosulfuron dose increased, indicating a decrease in the amount of active reaction centers and the rate of excess excitation energy consumption in the leaf. In this experiment, as the dose of nicosulfuron increased, the ABS/RC enhanced and the DI_O_/RC boosted, implying that the dissipation of the remaining active reaction centers expanded. This might be due to the increased burden on the remaining active reaction centers, compelling them to be more efficient in better dissipating the energy in the electron transfer chain.

### 4.4 Nicosulfuron increased ROS accumulation

In adverse circumstances, the content of ROS increases, and cell membranes are disrupted inside the plant. ROS causes oxidative damage to the photosynthetic apparatus in chloroplasts, resulting in the photoinhibition of PSII (Ajithkumar and Panneerselvam, 2014; Alzandi and Naguib, 2020). The study showed that under nicosulfuron stress, the generation rate of ${\text{O}}_{2}^{-}$ and H_2_O_2_ contents of sugar beet were considerably enhanced, along with MDA content and EL, which indicated significant oxidative damage to sugar beet. This might be due to the accelerated generation rate of ${\text{O}}_{2}^{-}$ in plants under adversity and the reduced ability of plants to utilize photosynthetic excitation energy. Excess electrons in the excited state synthesized electron transport chains to scavenge free ${\text{O}}_{2}^{-}$ . ROS triggered membrane lipid peroxidation and produced MDA, which altered the structure and function of cell membranes and disrupted membrane stability (Lanza and Dos Reis, 2021), thus leading to a significant increase in EL.

It was found that toxic treatment increased the activities of SOD and CAT in leaf to stable ROS content within a certain toxic concentration range (Wu et al., 2020). SOD, POD, and CAT activities enhanced afterward and decreased with increasing doses of nicosulfuron at 6 g a.i. ha^–1^, while APX activity gradually decreased. This may be because when ROS in plants exceeded the capacity of antioxidant enzymes, the antioxidant enzyme system cannot scavenged ROS in time. Excess ROS might decreased antioxidant enzyme activity, making plant cells more susceptible to oxidative damage (Li et al., 2011; Meloni and Martínez, 2021). In addition, plants can respond to toxic damage by regulating hormone levels to affect key enzyme activities in plants. Studies have shown that topical application of salicylic acid to valerian can reduce the toxic effects of bentazon herbicides by enhancing oxidative defense mechanisms and altering POD, CAT and APX enzyme activities (Khatooni et al., 2022).

## 5 Conclusion

Nicosulfuron led to the disruption in the function of PSII in sugar beet leaf. Photosynthetic parameters were altered, resulting in lower photosynthetic efficiency and significant photoinhibition. The ROS content, MDA content and EL of sugar beet leaf were enhanced significantly. The oxidative defense system of sugar beet was disrupted, and SOD, POD, CAT, and APX enzyme activities were inactivated considerably. The GR_50_ of nicosulfuron toxicity on sugar beet was 81.83 g a.i. ha^–1^. This study showed how nicosulfuron affected sugar beet. It also showed that the toxicity of nicosulfuron on sugar beet is a cause for concern and that the risk of herbicide in agricultural ecosystems should be taken into account.

## Data availability statement

The raw data supporting the conclusions of this article will be made available by the authors without undue reservation.

## Author contributions

LW: investigation, validation, formal analysis, and writing-Original draft preparation. MR: validation and writing-reviewing. BS: conceptualization, resources, supervision, and writing-reviewing. XS: writing-reviewing. WH: validation. XB: conceptualization, resources, supervision, and writing-reviewing. XZ: writing-reviewing. All authors contributed to the article and approved the submitted version.

## Funding

This work was supported by the China Agriculture Research System of MOF and MARA (CARS-170204), and the Science and Technology Department of Xinjiang Uygur Autonomous Region (2202E02006).

## Conflict of interest

The authors declare that the research was conducted in the absence of any commercial or financial relationships that could be construed as a potential conflict of interest.

## Publisher’s note

All claims expressed in this article are solely those of the authors and do not necessarily represent those of their affiliated organizations, or those of the publisher, the editors and the reviewers. Any product that may be evaluated in this article, or claim that may be made by its manufacturer, is not guaranteed or endorsed by the publisher.
